# Cost-effectiveness of Oral Protease Inhibitors Co-administration versus Pegylated Interferon-Α2b and Ribavirin Only for the Patients with Hepatitis C Genotype 1 in Kazakhstan Health Care Settings

**Published:** 2018-12

**Authors:** Alima ALMADIYEVA, Serik IBRAYEV, Assiya TURGAMBAYEVA, Alexandr KOSTYUK, Zayituna KHISMETOVA, Zhanar AKHMETOVA

**Affiliations:** 1. Dept. of Public Health, Astana Medical University, Astana, Kazakhstan; 2. National Center for Medicines, Medical Devices and Medical Equipment Expertise, Astana, Kazakhstan; 3. Dept. of Public Health, Semey State Medical University, Semey, Kazakhstan

**Keywords:** Hepatitis C, Antiviral treatment, Protease inhibitors, Cost-effectiveness

## Abstract

**Background:**

The triple therapy including peginterferon, ribavirin and protease inhibitors was more effective compared to the combination of only peginterferon and ribavirin. This study aimed to assess the cost-effectiveness of triple treatment in either treatment-naïve and treatment-experienced patients in Kazakhstan.

**Methods:**

A Markov model was created to assess long-term clinical advantages and the cost-effectiveness of the triple therapy from Kazakhstan payer perspective. Health state transition probabilities, pharmaceutical and other costs (according to the price in 2015), and utility rate were acquired from the published studies and publicly available sources. All used costs and benefits were discounted at 5% per year.

**Results:**

Despite treatment background, the patients, receiving boceprevir and telaprevir, were estimated to experience less serious liver-disease complications, more life-years, and more QALYs compared to the patients having standard of care. For treatment-experienced group, boceprevir and telaprevir were dominant, with more QALYs. For all the groups of patients, incremental costs per QALY gained were between USD14995 and USD18075. The total average cost of boceprevir is slightly more costly than a standard duration of treatment with telaprevir, and so is the average cost per SVR. Extensive sensitivity analyses verified robust model results.

**Conclusion:**

The inclusion of protease inhibitors to standard management for the therapy of patients with genotype 1 chronic HCV infection in Kazakhstan is predicted to be cost-effective using a typically applied willingness to pay threshold of USD37805 (3 times GDP per capita).

## Introduction

Chronic hepatitis C virus (HCV) infection as a major public health threat is a major cause of chronic liver disease, liver disease-related deaths (LRD), hepatocellular carcinoma (HC). Naturally, it is the most frequent sign for liver transplantation (1). About 130–170 million patients are equal to 3% of the world’s population infected with chronic HCV, so the burden of HCV is much, and the cost of managing HCV infection may differ from country to country (2–4). HCV prevalence in Kazakhstan is from 0.6% to 0.7%, which refers to about 60000–70000 HCV-infected patients. Genotype 1 (G1) is more frequent (55%) in Kazakhstan and the most difficult to treat among six main HCV genotypes (5).

Nowadays no vaccine against HCV exists; it is more effective to use the treatment pathways of decreasing the burden of the HCV infection. The primary objective of HCV treatment makes the virus undetectable for at least 6 months followed by the treatment that was known as a sustained virological response (SVR). The current clinical practiced to treat HCV infection is the combination of PEGylated interferon and ribavirin (PegIFN+RBV). The efficacy of double therapy depends on several factors like genotype, viral load, ethnic background, age, sex, fibrosis score and previous therapy (6). Efficacy of double therapy is about 40% in treatment-naïve (TN) patients with G1 and just 22% in treatment-experienced (TE) patients with G1 (7).

Two direct-acting antivirals (DAA), Boceprevir (BPV) and Telaprevir (TPV) have recently demonstrated significantly better treatment outcomes than traditional PegIFN+RBV for the treatment of G1 HCV infection. Both the DAA were approved in Europe and USA for using in the triple therapy of G1 HCV infection. The inclusion of BPV and TPV in the combination of PegIFN+RBV indicates the significant improvement of the SVR rates. SPRINT-2 and RESPOND-2 clinical trials (8, 9) have shown the results that the SVR rate, gained by adding BPV to PegIFN+RBV, is equal to 67%–68% vs. 40% of TN patients and to 69%-66% vs. 21% in TE patients. ADVANCE and RELIAZE clinical trials (10, 11) have shown that the SVR rate gained by adding TPV to PR is 69%–75% vs. 44% in TN patients and 54%–59% vs. 15% in TE patients.

Therefore, the objective of our study was evaluation and comparison of the average cost per patient for BPV and TPV, and the cost per SVR using BPV, TPV, and standard of care (SOC) treatments as well as cost-effectiveness estimation in comparison with the latest clinical practice guidelines for the treatment of patients that have G1 HCV-infection in Kazakhstan.

## Materials and Methods

Sustained virological response facts as a determination of antiviral treatment effectiveness were taken from the multicenter, placebo-controlled trials (ADVANCE (10), RELIAZE (11), RESPOND-2 (9) and SPRINT-2 (8)). The clinical trials, during which METAVIR scoring was applied, have determined the severity of hepatitis C disease; but fibrosis scores reporting is not obligatory in several cases in Kazakhstan. We have applied the patients’ distribution by METAVIR scores according to the clinical trials (12, 13) because of a lack of available data about fibrosis scores distribution in Kazakhstan. We have carried out semi-structured interviews in 2015 to determine the average age of the patient’s cohort in the analysis. Three hundred thirty-seven patient reports from 14 centers were involved. Only complete therapy information was included in the analysis. The Kazakhstan HCV population took part in the study, which included 46% male and 54% female patients. The analysis has demonstrated that the average age of patients having HCV infection treated is 49.3 yr.

The progression rates of the normal chronic HCV infection were taken (14). The baseline probability of HC development in patients with F3 state was approximated (15). Total annual number of HC increases with progression of cirrhosis. An excessive risk of HC and DC from compensated cirrhosis was approximated (16, 17). The probability of liver transplantation necessity in advanced stages and mortality rates were derived from the Ministry of Health’s database investigation, analysis, and other published data (5). Adverse events such as anemia are a major reason that the patients decline and reduce the dosage or stop therapy altogether (18). The patients received therapy-related anaemia in the clinical trials (8–11) were managed by the decrease of ribavirin dose or anti-anemic therapy. In Kazakhstan, progressive ribavirin dose reduction is proposed as commonly used option for the management of HCV therapy-induced anemia (19). Accessible anti-anemic therapy reimbursement is indicated in the management of HCV therapy-related anemia under Kazakhstani conditions. Moreover, there is no significant difference in SVR rates achieved in anemic patients receiving PI plus PR and using ribavirin dose reduction or anti-anemic therapy as well (20). Usually, anemia cases are managed by ribavirin dose reduction in Kazakhstan therapeutical practice; however, different anti-anemic therapy costs were analyzed. The direct medical costs of HCV disease include the cost of therapy, HCV overall health state-related costs as well as including the cost of liver transplantation. QALYs indicator is used regardless of whether the patient was treated or not, and for untreated patients, in particular, who have the disease progression and quantity of time spent in each of the HCV infection stages. All the costs and QALYs were discounted at 5%.

We have forecasted the lifetime incidence of complications, total costs, and QALYs related to each management strategy. We also projected the incremental cost-effectiveness ratios (ICERs) for BPV-based and TPV-based regimens compared to PR treatment. The model has evaluated the cost-effectiveness of BPV-based and TPV-based therapy separately for TN and TE groups. We have calculated direct medical costs including drug acquisition costs that would be incurred by the healthcare payer in addition to medical cost offsets and adverse event costs (including treatment-related anemia). Indirect costs due to lost productivity were not included. Presently there is no any official cost-effectiveness threshold in Kazakhstan. Therefore, WHO-CHOICE (21) guideline around the cost-effectiveness threshold (3 times of gross domestic product (GDP) per capita) was taken into consideration when interpreting the benefits. Based on the 2015 World Bank data, 3 times of GDP per capita is USD 37 805.

**Fig. 1:**
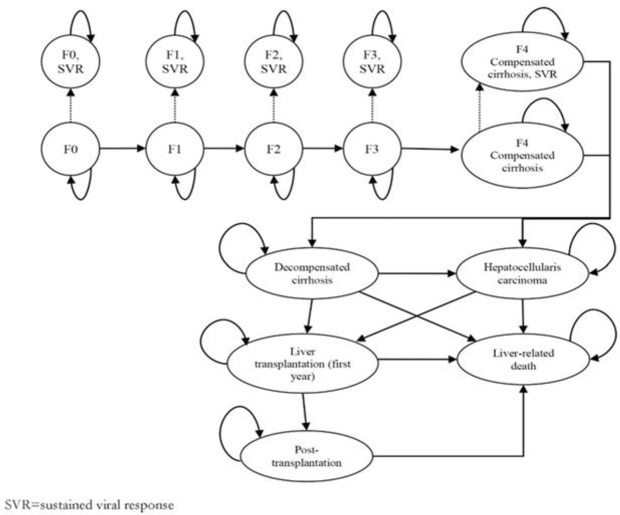
Markov state-transition model diagram

A constructed model simulates DAA-based therapy tactics and standard double therapy, permitted by the Ministry of Health of the Republic of Kazakhstan (MoH). Patients, not achieved the Hepatitis C virus indication in the blood, were shown in a Markov state-transition model diagram (Fig. 1) as treatment-naïve (TN) as well as we indicated treatment-experienced patients (TE), already undergone any antivirus therapy. We used Markov model for analysis of the cost per patient for the treatment course and follow-up duration with the 4-wk intervals for the first 48 wk. Consider that the next 24 wk (follow-up time) as a one-time interval. Our model also used adverse event data, i.e., the proportion of patients, expected to experience adverse events common to PI and PR treatment (i.e., anemia, neutropenia, rash, and pruritus), taken from the indirect comparison by Cooper et al (22). The model design indicates the incidence of advanced liver disease (decompensated cirrhosis (DC), HC and liver transplantation. Therefore, it estimates the health outcomes and costs of various treatment approaches over the lifetime horizon in cohorts of patients with HCV in Kazakhstan. All the costs were calculated in Kazakhstan Tenge (KZT) at the exchange rate of US dollar (USD) in 2015 (1USD = 339,986KZT).

To analyze the robustness of the model outcomes and impact of varying factors on costs and QALYs, both deterministic and probabilistic sensitivity analyses (PSA) have been conducted. Factors such as age, transition probabilities, treatment effectiveness, quality of life weights, health care costs, discount rates, implication of adverse events cost and mortality in health state of compensated cirrhosis were analyzed over a possible range. The base case input data and the lower- and upper boundaries of ranges were extracted from the published data while available. In the basic case scenario, all the costs and health outcomes were discounted at 5%, as specified in the Kazakhstan Health Technology Assessment Guidelines on processing health economic studies (23). Using the guideline, the sensitivity analysis was also completed based on the discount rates of costs and health outcomes applying the range of 3%–6% and 0%–6%, respectively.

## Results

### Base-case Analysis

In TN patients, BPV-based triple combination treatment has predicted an increase in the life expectancy by 0.98 yr and QALY by 0.59 comparing to treatment with SOC treatment (Table 1).

**Table 1:** Cost-effectiveness outcomes, USD 2015

***Strategy***	***Life Expectancy (years)***		***Cost (USD)***		***QALY***		***ICER (per QALY) (USD)***	
	***TN***	***TE***	***TN***	***TE***	***TN***	***TE***	***TN***	***TE***
SOC	25.31	23.77	7 489	4 913	10.96	10.35		
BPV	26.29	26.19	17 166	21 857	11.55	11.48	16 403	14 995
∆BPV	0.98	2.42	9 678	16 944	0.59	1.13		
TPV	26.71	26.39	25 564	27 644	11.96	11.75	18 075	16 237
∆TPV	1.40	2.62	18 075	22 732	1.00	1.4		

Abbreviation: SOC=standard of care (only PR); BPV=boceprevir added to PR, TPV=telaprevir added to PR; TN=treatment-naïve patients; TE=treatment experienced patients

TPV-based triple combination treatment has predicted an increase in the life expectancy by 1.40 yr and QALY by 1.00 comparing to treatment with SOC treatment. BPV-based treatment costs amounted to USD 9678 which are more than SOC, TPV-based treatment costs amounted to USD 18075 which are more than SOC.

In TE patients, BPV-based treatment has predicted an increase in the life expectancy by 2.42 yr and QALYs by 1.13, and the cost amounted to USD 16944, which is more compared to the therapy with SOC. BPV triple treatment has resulted in USD 16403 and USD 14995 per QALY in TN and TE groups, correspondingly (Table 1). TPV triple treatment has resulted in the costs, which amounted to USD18075 and USD16237 per QALY in TN and TE groups, correspondingly. The relative risk of severe liver disease-related complications, such as DC, HC and LRD, was predicted to decrease over lifetime equally in TN and TE patients. Table 2 illustrates the total costs and cost per SVR for standard-duration BPV and TPV for both TN and experienced patients, as derived from our budget impact analysis model. Based on it, the total average cost of standard BPV «arm» duration is USD 17166 for TN patients and USD 21857 for TE patients. Comparison of the total average cost with standard TPV «arm» duration, which is USD 25564 for TN patients and USD 27644 for TE patients. The cost per SVR with standard BPV duration regimen is USD26750 for TN patients and USD35396 for TE patients. Comparison of the total average cost of SVR having standard TPV duration, which is USD 39835 for TN patients and USD 43699 for TE persons.

**Table 2:** Median cost estimates from the budget impact analysis for HCV therapy, USD 2015

	***Average TN***	***TN non-cirrhotic: early responder***	***TN non-cirrhotic: late responder***	***TN cirrhotic***	***Average TE***	***TE non-cirrhotic: early responder***	***TE non-cirrhotic: late responder***	***TE cirrhotic***
BPV arm								
PR	5 289	4 956	5 591	6 653	6 125	6 555	5 090	7 565
BPV	10 549	11 317	8 945	15 880	13 970	15 619	9 267	18 333
Clinical monitoring	147	141	154	169	158	158	148	182
AE management	1 182	910	1 478	1 832	1 603	1 624	1 330	2 320
Total cost	17 166	17 324	16 169	24 534	21 857	23 957	15 835	28 399
Assumed SVR, %	64.2	89.0	33.0	43.0	61.8	90.0	36	77.0
Cost per SVR	26 750	19 465	48 996	57 055	35 396	26 619	43 987	36 881
TPV arm								
PR	4 936	4 313	5 591	6 653	5 823	4 470	5 412	7 565
TPV	19 371	20 194	18 167	20 261	20 083	20 261	18 775	19 586
Clinical monitoring	145	136	154	169	158	138	153	182
AE management	1 112	702	1 575	1 952	1 581	743	1 577	2 472
Total cost	25 564	25 345	25 488	29 034	27 644	25 612	25 917	29 804
Assumed SVR, %	64.2	89.0	33.0	43.0	63.3	94.0	46.0	77.0
Cost per SVR	39 835	28 477	77 235	67 522	43 699	27 247	56 341	38 706

Abbreviations: TN – treatment-naïve; TE – treatment-experienced; PR - peginterferon plus ribavirin; BPV - boceprevir; TPV - telaprevir, BPV arm - PR + BPV; TPV arm - PR + TPV; AE, adverse event; SOC, standard of care; SVR, sustained virological response

The total average cost of standard treatment duration of using BPV is slightly cheaper than standard treatment duration of using TPV and, thus, it is the average cost per SVR. In addition, the total average cost of HCV therapy with BPV is significantly smaller than HCV therapy with TPV and, thus, it is the average cost per SVR. Additionally, the cost per SVR is comparable to HCV therapy using BPV and SOC, while the cost per SVR is more costly with TPV regimen.

The observed differences can be defined by different scores where the costs are accumulated in the framework of different treatments. In TPV arm, all the patients receive expensive part of treatment within the first 12 wk. In BPV arm, the patients take only SOC for the first 4 wk of the therapy course, whereas BPV, the most expensive component of treatment, is administered throughout the remaining 44 wk. The discontinuation rates, and, thus, the proportion of patients, staying on treatment, play a role in the cost of BPV and TPV treatment as well. Most patients continue for 12 wk of the full 48-week therapy course, and, consequently, all of them receive a full course of TPV. By comparison, the patients, who have been discontinuing only between 12 and 48 wk, were going to receive a portion of the full course of BPV. Therefore, the total average costs are usually pulled in the direction of BPV favoring, because a full course of therapy is not wasted on those patients who discontinue.

The costs related to clinical monitoring and AE management are both relatively small compared with the costs of SOC and BPV/TPV, and, thus, have a small relative impact on total average cost estimations.

### Sensitivity Analyses

The deterministic sensitivity analysis (Table 3) demonstrates that the model outcomes are sensitive to changes in factors of efficacy (SVR rates), utilities and transition probabilities. The ICER, received from the model, exceeded the cost-effectiveness threshold only when assuming the lower value of transition probabilities or the lower incremental health gain (SVR), achieved by BPV and TPV arms.

**Table 3:** Deterministic Sensitivity Analysis Outcomes, ICER (USD)

***Parameter***	***BPV arm costs, USD 2015***		***TPV arm costs, USD 2015***	
	***Treatment-naive***	***Treatment-experienced***	***Treatment-naive***	***Treatment-experienced***
Base-case	16 403	14 995	18 075	16 237
Age (average age of cohort)				
35 yr old	11 556	10 685	12 734	11 570
45 yr old	14 159	12 980	15 602	14 055
55 yr old	19 323	17 749	21 293	19 219
Probabilities of Receiving Liver Transplantation				
DC: 0.032; HC: 0.016	16 360	14 919	18 028	16 154
Discount Rate				
0%	5 312	4 718	5 854	5 109
3%	11 154	10 048	12 291	10 880
Costs: 5%; Outcome: 0%	6 180	5 585	6 810	6 047
Progression after SVR (DC: 0,008; HC: 0,005^*^	16 445	16 908	18 121	18 308
Transition Probabilities				
All lower limits	22 138	22 034	24 395	23 858
All upper limits	12 106	12 827	13 340	13 890
Compensated cirrhosis →Death (0,0566^**^	13 503	11 501	14 880	12 454
Health State Costs				
−15%	16 636	15 352	18 331	16 623
+15%	16 170	14 689	17 818	15 906
Utilities				
All lower limits	18 181	16 500	20 034	17 866
All upper limits	12 530	12 037	13 807	13 034
SVR				
Low 95%	22 244	19 687	24 512	21 318
High 95%	12 847	11 986	14 157	12 978

Abbreviation: SOC=standard of care (only PR; BPV=boceprevir added to PR, TPV=telaprevir added to PR; SVR=sustained virological response

The BPV-based regimen was cost-effective with a probability of 44% and 40% in TN and TE people respectively, and the TPV-based regimen was cost-effective with a probability of 48% and 43% in TN and TE groups respectively, at a willingness-to-pay value of USD 37805.

## Discussion

Clinical trial results (ADVANCE, RELIAZE, RESPOND-2, SPRINT-2) have demonstrated that PI (BPV and TPV)-based triple treatment insured significantly more effective management to the patients with G1 chronic HCV disease compared with current standard-of-care therapy. The outcomes of our model have shown that PI in addition to PR, according to the estimates, is a cost-effective therapy strategy compared with PR double combination for each TN and TE patients with applying a willingness-to-pay threshold of USD37805.

In TN patients, the cost of BPV-based and TPV-based tactics are lower than in TE patients – USD 9678 vs. USD 16944 and USD 18075 vs. USD 22732, correspondingly. This can be defined by significantly shorter length of the treatment (28-week or 24-week vs. 48-week) in non-cirrhotic TN patients who had a rapid virologic response. However, bigger additional cost in TE patients was counterweighed by bigger incremental health gains (QALYs) in TE patients comparing to TN patients. Therefore, BPV-based and TPV-based strategies have resulted in smaller ICER in TE patients rather than in TN patients.

We have performed evaluation of the total average cost and cost per SVR for HCV therapy with BPV and TPV, added to SOC and for SOC alone. Our results show that HCV therapy with BPV is significantly less costly than HCV therapy with TPV and that the cost per SVR for HCV therapy with BPV is comparable with the cost per SVR for SOC.

Our study has several strengths and limitations. We did not include the opportunity of spontaneous HCV clearance in our model, observed in people with mild states (F0 and F1) of HCV. The patients, achieved SVR, were definitely not at risk for reactivation of HCV infection. Long-term outcomes of various studies (24) in HCV infection demonstrate that more than 90% of people, achieved SVR, remained virus-free during a long-term follow-up. We used one of the strongest clinical evidence in the form of current results by indirect and multiple treatment comparative meta-analyses. We applied detailed discontinuation data of clinical trials, but current clinical evidence is still staying limited since only several studies for every of the considered treatment regimens and patient groups (naïve and experienced) exist. For adverse events modeling, a constant relative risk within every 4-week interval was used. However, probably, it might happen with varying risks during the full course of treatment.

Sensitivity analysis has been also conducted on the base-case age of the HCV-infected population. Nowadays, the average age of people with HCV infection is 49.3 yr. The outcomes let us suggest that BPV is more beneficial (cost-effective) therapeutical tactic of HCV treatment for young patients in comparison with the base-case population.

Our results might lead to convenient implications for clinical practice. If its forecasts that HCV therapy with BPV and TPV, added to SOC, is more effective compared to SOC, it might appear beneficial from the societal perspective and for the patients. Patients generally prefer the most effective treatments, i.e., BPV or TPV rather than SOC. However, funding bodies should make decisions on that whether they should pay an extra money for the extra gain in the effectiveness. In this scenario, the cost of treating one person (by SVR) is comparable with SOC, the same as BPV or TPV added to SOC. This is a condition for each TN and TE patient.

Thus, a higher level of efficiency and safety in the treatment of HCV G1 has shown interferon-free various modes of the therapy, reflected in new EASL recommendations (25). Moreover, in the view of the limited resources of health as well as the presence of specific contraindications of interferon-free therapy, using of BPV and TPV remains an important cost-effective option in the context of Kazakhstan.

## Conclusion

The increased SVR rates, detected for BPV and TPV, compared with SOC alone, led to fewer HCV-related complications and LRD, likewise as improving the survival and quality-adjusted survival in the patient groups. Within the limitations of our model, BPV and TPV were estimated to be a cost-effective or cost-saving treatment option compared with SOC for the treatment of adults with chronic G1 HCV infection and compensated liver disease from the point of Kazakhstan payers view. Moreover, our model expects that high pharmaceutical costs of HCV-treatment for the patients, using BPV or TPV, compared with SOC alone, should be offset generally or completely, depending on the patient group, by savings in medical costs related to liver-disease complications.

## Ethical considerations

Ethical issues (Including plagiarism, informed consent, misconduct, data fabrication and/or falsification, double publication and/or submission, redundancy, etc.) have been completely observed by the authors.
